# Silencing of PTK7 in Colon Cancer Cells: Caspase-10-Dependent Apoptosis via Mitochondrial Pathway

**DOI:** 10.1371/journal.pone.0014018

**Published:** 2010-11-16

**Authors:** Ling Meng, Kwame Sefah, Meghan B. O'Donoghue, Guizhi Zhu, Dihua Shangguan, Afshan Noorali, Yan Chen, Lei Zhou, Weihong Tan

**Affiliations:** Department of Chemistry and Department of Physiology and Functional Genomics, Shands Cancer Center and Center for Research at the Bio/Nano Interface, UF Genetics Institute and McKnight Brain Institute, University of Florida, Gainesville, Florida, United States of America; The University of Hong Kong, Hong Kong

## Abstract

Protein tyrosine kinase-7 (PTK7) is a catalytically inactive receptor tyrosine kinase (RTK). PTK7 is upregulated in many common human cancers, including colon cancer, lung cancer, gastric cancer and acute myeloid leukemia. The reason for this up-regulation is not yet known. To explore the functional role of PTK7, the expression of PTK7 in HCT 116 cells was examined using small interference (siRNA)-mediated gene silencing. Following transfection, the siRNA successfully suppressed PTK7 mRNA and protein expression. Knocking down of PTK7 in HCT 116 cells inhibited cell proliferation compared to control groups and induced apoptosis. Furthermore, this apoptosis was characterized by decreased mitochondrial membrane potential and activation of caspase-9 and -10. Addition of a caspase-10 inhibitor totally blocked this apoptosis, suggesting that caspase-10 may play a critical role in PTK7-knockdown-induced apoptosis, downstream of mitochondria. These observations may indicate a role for PTK7 in cell proliferation and cell apoptosis and may provide a potential therapeutic pathway for the treatment of a variety of cancers.

## Introduction

Receptor tyrosine kinases (RTKs) compose a class of transmembrane signaling proteins that transmit extracellular signals to the interior of the cell. Misregulation of RTKs plays an important role in the development and/or progression of many forms of cancer [1]. Protein tyrosine kinase-7 (PTK7), which is also known as colon carcinoma kinase-4 (CCK4), is a relatively new and little studied member of the RTK superfamily. It contains an extracellular domain with seven immunoglobulin-like loops, a transmembrane domain, and a catalytically inactive tyrosine kinase domain [2], [3]. However, as a result of an amino acid substitution within the catalytic domain, PTK7 is a pseudokinase without detectable catalytic tyrosine kinase activity [1], [4]. It was originally identified as a gene-expressed colon cancer-derived cell line, but it is not expressed in human adult colon tissues [2]. In contrast, high levels of PTK7 expression are seen in fetal mouse colons [1], [2], [4]. The expression of PTK7 is up-regulated in many common human cancers, including colon cancer, lung cancer, gastric cancer and acute myeloid leukemia [2], [5], [6], [7], [8], [9]. Recently, PTK7 was identified as a novel regulator of non-canonical WNT or planar cell polarity (PCP) signaling [10], [11]. PTK7 also appears to play an important role in tube formation, migration, invasion of endothelia and angiogenesis in HUVEC cells [12]. However, the functional role of PTK7 in cell proliferation and apoptosis remains unclear.

Aptotosis is programmed cell death, typically mediated by a family of cysteine proteases known as caspases [13]. Caspases are synthesized as inactive proenzymes with either a long (caspase-8, -9 and -10) or a short (caspase-3, -6 and -7) prodomain [14], [15]. These latter proteases cleave a series of essential intracellular proteins leading to cell death [16].

Two main apoptosis pathways have been identified. The intrinsic pathway (mitochondria pathway) involves a decrease in mitochondrial membrane potential and release of cytochrome *c* [17], which activates caspase-9 through the apoptosome. Then, caspase-9 initiates a proteolytic caspase cascade that kills the cells. The extrinsic pathway (death receptor pathway) involves the tumor necrosis factor (TNF) receptor superfamily. In response to TNF ligand binding, these membrane receptors recruit adapter molecules and activate caspase-8 in the death-inducing signaling complex (DISC). Activated caspase-8 either directly activates downstream effector caspases, such as caspase-3, or connects to the intrinsic pathway through cleavage of BCL-2 Interacting Domain (Bid) to truncated Bid (tBid)[18].

The caspase-10 gene is linked to the caspase-8 gene at the human chromosome locus 2q33-34 [19]. However, the physiological functions of caspase-10 remain poorly understood, although it is thought to play a role in the death receptor pathway. Caspase-10 was also reported to be activated downstream of the mitochondria in cytotoxic drug-induced apoptosis of tumor cells [20]. Acquired inactivating mutations of caspase-10 have been identified in tumor cells from patients with solid tumors [21], [22], [23]. Recently, caspase-10 was shown to play a role in apoptosis induced by paclitaxel, an anticancer drug, through a Fas-Associated protein with Death Domain (FADD) -dependent mechanism [24].

The term RNA interference (RNAi) was first used by Fire *et al.* [25] in their work on *Caenorhabditis elegans*. RNAi is a cellular mechanism by which small interfering RNAs (siRNAs) (19-23 nucleotides in length) trigger the degradation of specific mRNA [25], [26]. It has been demonstrated that siRNAs can silence cognate gene expression via the RNAi pathway in mammalian cells [27]. The properties of RNAi, including stringent target-gene specificity and simplicity of design and testing [28], have greatly widened its potential for mechanistic studies of proteins, as well as for therapeutic approaches to treat diseases, including cancer [29], [30].

In this study, a siRNA targeting human PTK7 mRNA was used for maximal inhibition of PTK7 expression in order to probe the role of PTK7 in apoptosis and proliferation. Knocking down PTK7 in HCT 116 cells inhibited cell proliferation and induced apoptosis. Furthermore, this apoptosis was characterized by decreased mitochondrial membrane potential and activation of caspase-9 and -10. Addition of a caspase-10 inhibitor totally blocked this apoptosis, suggesting that caspase-10 may play a critical role in PTK7-knockdown-induced apoptosis downstream of mitochondria. Therefore, these observations may indicate a role for PTK7 in cell proliferation and cell apoptosis.

## Materials and Methods

### Materials

McCoy's 5A media were purchased from ATCC; fetal bovine serum (FBS) (heat inactivated) was purchased from GIBCO, and penicillin-streptomycin was purchased from Cellgro. Micro-FastTrack 2.0 Kit was purchased from Invitrogen. A colorimetric bromodeoxyuridine (BrdU) kit was from BD Pharmingen. IScript One-Step RT-PCR Kit with SYBR Green was from Biorad. Protease inhibitor cocktail (mixture of 4-(2-aminoethyl)benzenesulfonyl fluoride (AEBSF), E-64, bestatin, leupeptin, aprotinin, and sodium EDTA) and 0.4% trypan blue were from Sigma. The protein assay kit was from Bio-Rad. Antibodies against caspase-9 and β-actin were from Cell Signaling Technology. Antibody against caspase-10 was from Millipore. Antibody against PTK7 was from Abnova. Goat anti-mouse IgG HRP-conjugated secondary antibody and goat anti-rabbit IgG HRP-conjugated secondary antibody were purchased from Cell Signaling Technology. Vybrant Apoptosis Assay Kit #2, 4× NuPAGE LDS sample buffer, 4–12% NuPAGE Bis-Tris gels, 20× NuPAGE MOPS SDS running buffer, and 20× NuPAGE transfer buffer were from Invitrogen. Immobilon-P transfer membrane was from Millipore. SuperSignal West Dura Extended-Duration Substrate and Restore plus Western blot Stripping buffer were from Thermo Scientific. X-ray films were from ISCBioExpress. JC-1 (5,5′,6,6′-tetrachloro-1,1′,3,3′- tetraethylbenzimidazolylcarbocyanine iodide) was purchased from Anaspec. Caspase-9 inhibitor (Z-LEHD-FMK), caspase-3 inhibitor (Z-DEVD-FMK), caspase-8 inhibitor (Z-IETD-FMK), caspase-family inhibitor (Z-VAD-FMK), caspase-1 inhibitor (Z-YVAD-FMK), caspase-10 inhibitor (Z-AEVD-FMK) and caspase-2 inhibitor (Z-VDVAD-FMK) were purchased from BioVision. Caspase-10 Fluorometric Protease Assay Kit was from Millipore.

### Cell Culture

HCT 116 (colon carcinoma) cells were obtained from ATCC (Manassas, VA) and maintained in tissue culture at 37°C and 5% CO_2_. p53-null HCT 116 cells were provided by Dr. Bert Vogelstein (The Johns Hopkins Kimmel Cancer Center). Cells were cultured in McCoy's 5A medium supplemented with 10% fetal bovine serum (FBS) (heat inactivated) and 100 IU/mL penicillin-streptomycin.

### RNA interference

HiPerFect transfection reagent, HP-validated siRNA specific for PTK7, named PTK7 siRNA (sense: 5′- CGGGATGATGTCACTGGAGAA-3′), and a nonspecific siRNA (AllStars Negative Control siRNA) were purchased from Qiagen. HCT 116 cells were transfected with siRNA by HiPerFect transfection reagent. On day 1, cells in exponential growth phase were harvested and suspended in growth medium. Cells were divided into four groups and were treated with PTK7 siRNA, a nonspecific siRNA as negative control, HiPerFect vehicle only, or were left untreated. For each transfection, a 500 µL cell suspension was transfected with 25 nM siRNA using 4 µL transfection reagent in 24-well plates. Cells were kept in normal culture conditions and collected 2, 3 or 4 days after transfection for analysis.

### Flow Cytometric Analysis

After transfection with siRNAs as described above, cells were trypsinized, washed twice in PBS, and counted using a hemocytometer. Aliquots of 5×10^5^ cells were incubated with excess phycoerythring (PE)-labeled anti-PTK7 in 200 µL of binding buffer (PBS containing 5 mM MgCl_2,_ 4.5 mg/mL glucose, 0.1 mg/mL yeast tRNA, 1 mg/mL BSA and 20% FBS) on ice for 30 min. Cells were then washed twice with 1 mL of binding buffer and suspended in 0.3 mL of binding buffer. The fluorescence was determined with a FACScan cytometer (Becton Dickinson Immunocytometry Systems, San Jose, CA). PE-labeled anti-IgG was used as a negative control.

### Quantitative RT-PCR

Total mRNA from cells treated with various siRNAs was extracted with Micro-FastTrack 2.0 Kit according to the manufacturer's instructions. Real-time PCR was performed on mRNA (50 ng) with iScript One-Step RT-PCR Kit using SYBR Green with a Biorad iCycler. All reactions were performed in a 50-µL volume in triplicate. Primers for human PTK7 were purchased from Qiagen (QT00015568). PCR parameters were as follows: 50°C for 30 min, 5 min of Taq activation at 95°C, followed by 45 cycles of PCR: 95°C×30 s, 57°C×60 s, and 72°C×60 s. The relative amount of target mRNA was normalized to GAPDH mRNA. Specificity was verified by melting curve analysis. Means and standard errors of at least three replicates of each experiment are calculated. Significance was determined by t-test, a p value≤0.05 indicated by an asterisk.

### Cell Number detection by Trypan Blue Exclusion Assay

For four days after treatment, cell suspensions were prepared by trypsinization, and cells were resuspended in 1 mL media. To 25 µL of the cell suspension, 25 µL of 0.4% trypan blue was added, and cells were counted using a hemocytometer.

### Proliferation Assay

Cell proliferation was studied using a colorimetric bromodeoxyuridine (BrdU) kit according to the manufacturer's instructions. First, cells were transfected with siRNA. After 48 h of treatment, 10 µM BrdU solution was added to the medium. The medium was discarded after 2 h, and cells were fixed and permeabilized with BD Cytofix/Cytoperm Buffer for 30 min at room temperature. After removing Cytofix/Cytoperm Buffer, cells were incubated with 100 µL of diluted DNase (diluted to 300 µg/mL in PBS) for 1 hour at 37°C to expose incorporated BrdU. Cells were then resuspended in 50 µL of BD Perm/Wash Buffer containing diluted FITC-labeled anti-BrdU and incubated for 20 minutes at room temperature. Finally, cells were incubated with 20 µL of the 7-AAD solution. Samples were analyzed by flow cytometry. Means and standard errors of at least three replicates of each experiment were calculated. Significance was determined by t-test; a p value≤0.05 is indicated by an asterisk.

### Annexin V/Propidium Iodide Double-Staining Assay

Annexin V/propidium iodide (PI) double-staining was performed using the Invitrogen Vybrant Apoptosis Assay Kit #2. Cells were washed twice in ice-cold PBS buffer and centrifuged at 900 rpm for 3 min. The pellets were resuspended in binding buffer at a density of 10^6^ cells/mL. A sample solution (100 µL) was double-stained with 5 µL Annexin V/Alexa Fluor 488 and 2 µL 100 µg/mL PI. After incubation at room temperature for 15 min, 400 µL of binding buffer was added, and cells were analyzed by flow cytometry.

### Western Blot Analyses

After HCT 116 cells were transfected with PTK7 siRNA for 12 h, 24 h, 30 h, and 48 h, whole cells were harvested and washed twice with ice-cold PBS. Then cells were lysed in radioimmunoprecipitation buffer (150 mM NaCl, 1% Triton X-100, 1% sodium deoxycholate, 0.1% sodium dodecyl sulfate (SDS), 50 mM Tris-HCl, pH 7.5, and 2 mM EDTA) in the presence of proteinase inhibitor cocktail for 20 min on ice. Lysates were centrifuged at 14,000 rpm for 20 min at 4°C, and the protein content in the supernatant was measured using the Bio-Rad protein assay. Fifty micrograms of supernatant proteins were mixed with 4× NuPAGE LDS sample buffer and heated at 70°C for 10 min. The proteins were separated on 4–12% NuPAGE Bis-Tris gels with 1× NuPAGE MOPS SDS running buffer and then electrotransferred onto a PVDF transfer membrane with NuPAGE transfer buffer at 50 V for 1 h. The membranes were blocked with 5% nonfat dry milk in PBS buffer containing 0.2% Tween 20 (PBST) for 2 h at room temperature. The membranes were probed with primary antibodies in PBST containing 5% nonfat dry milk overnight at 4°C. After three successive washings with PBST for 10 min, the membranes were incubated with horseradish peroxidase-conjugated goat anti-mouse IgG antibody or goat anti-rabbit IgG antibody in PBST containing 5% nonfat dry milk for 1 h at room temperature. After three successive washings with PBST for 10 min, the proteins signals were developed with a SuperSignal West Dura Extended Duration Substrate kit and transferred from the membrane to X-ray films. Protein loading was normalized by probing the same membrane with anti-actin antibody. For β-actin detection, previously used membranes were soaked in Restore Plus Western Blot Stripping Buffer at room temperature for 30 min and hybridized with anti-β actin.

### Measurement of Mitochondrial Membrane Potential

Dye JC-1 (5,5′,6,6′-tetrachloro-1,1′,3,3′- tetraethylbenzimidazolylcarbocyanine iodide) was used to determine mitochondrial membrane potential (ΔΨ_m_), the loss of which is regarded as a crucial step in the apoptosis pathway. HCT 116 cells were transfected with siRNA for 48 h or 72 h, after which the cells were washed with cold PBS and stained by incubating with 2 µM JC-1 for 20 min at 37°C. Then, the mitochondrial membrane potential was detected by fluorescence microscopy and flow cytometry at 590 nm.

### Caspase-10 Activity Measurement

Caspase-10 activity was measured using the Caspase-10 Fluorometic Protease Assay Kit. Briefly, cells were transfected with PTK7 siRNA, and after 24 h or 48 h cells were harvested. Two million cells were resuspended in chilled lysis buffer and incubated on ice for 10 min. Then, 50 µL of 2× Reaction Buffer and 5 µL of the 1 mM AEVD-AFC substrate (50 µM final concentration) were added to each sample. After incubation at 37°C for 2 h, samples were analyzed using a microplate reader equipped with a 400 nm excitation filter and a 505 nm emission filter. Means and standard errors of at least three replicates of each experiment were calculated. Significance was determined by t-test, a p value≤0.05 is indicated by an asterisk.

## Results

### Inhibition of PTK7 expression by PTK7 siRNA

**Figure 1:**
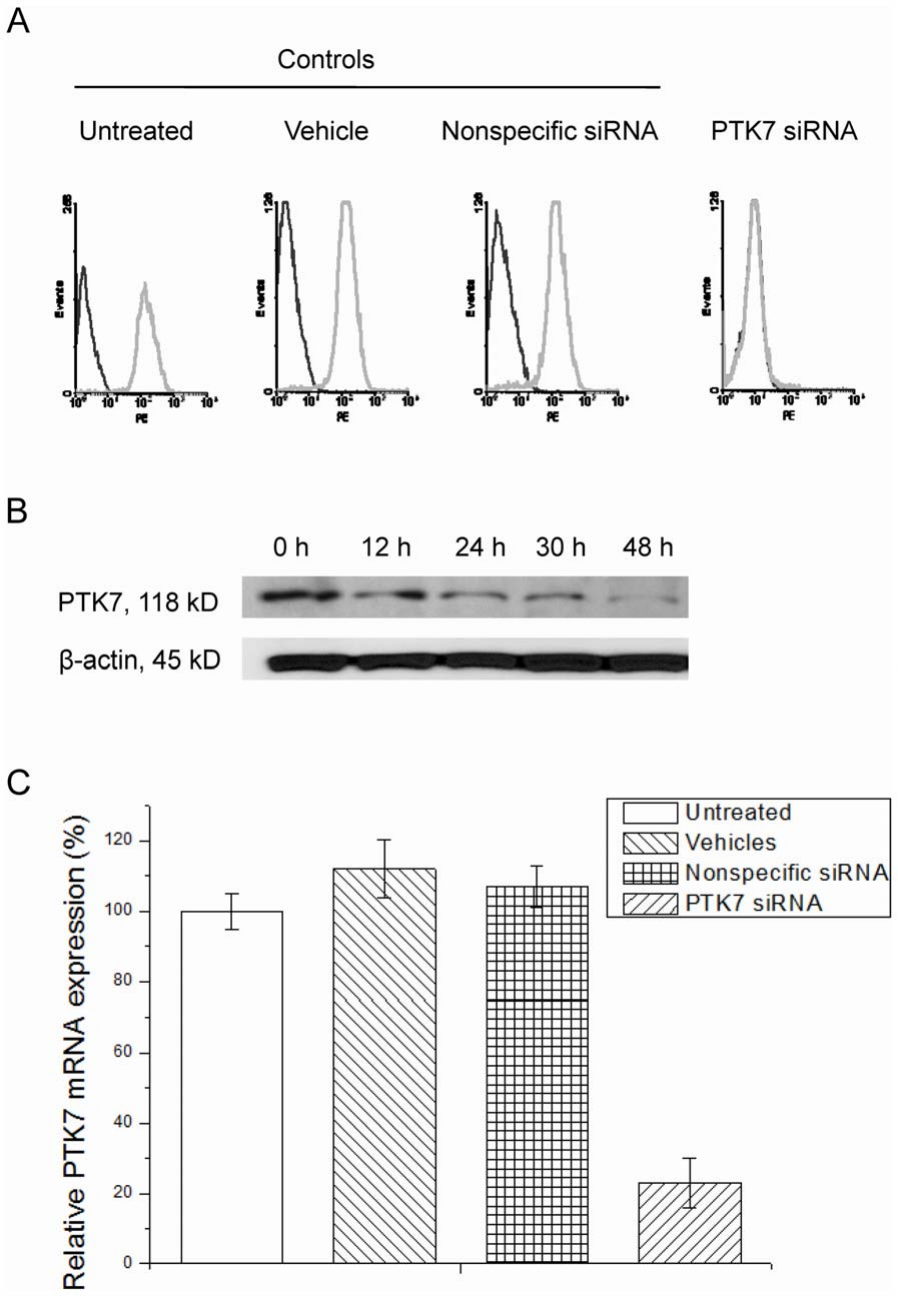
PTK7 expression in HCT 116 cells after treatment with vehicle, nonspecific siRNA and PTK7 siRNA. (A) Flow cytometry assay for the binding of the PE-labeled anti-PTK7 with HCT 116 cells (Grey curves). The black curves represent the background binding of anti-IgG-PE. The concentration of the antibody in the binding buffer was 2 µg/µL. (B) Western blot analysis of PTK7 in HCT 116 cells transfected by PTK7 siRNAs. The membrane was stripped and reprobed by β-actin antibody as a loading control. (C) Suppression of PTK7 mRNA expression in HCT 116 cells by PTK7 siRNAs. Cells were harvested after 48 h of treatment. RT-PCR was performed using gene-specific primers. The amount of PTK7 mRNA expression was normalized to the untreated group. Data are mean±s.d. of three independent experiments. *Student's t-test: P<0.05.

Expression of PTK7 in HCT 116, human colon carcinoma cells, was investigated by flow cytometry (Fig. 1A, untreated) and Western blot (Fig. 1B, 0 h). Comparing the fluorescence signal of PE-labeled anti-PTK7 to PE-labeled anti-mouse IgG clearly shows that PTK7 is expressed in HCT 116 cells.

Expression of PTK7 was knocked down using PTK7-targeted siRNA and the flow cytometry results for the targeted cells were compared to those exposed to vehicle only, nonspecific siRNA or untreated, as shown in Fig. 1A. After 48 hours, the peak of anti-PTK7-PE in HCT 116 transfected with PTK7 siRNA shifted back to the peak of the background control protein, anti-IgG-PE, indicating that the PTK7 expression level in HCT 116 cells transfected with PTK7 siRNA greatly decreased. At the same time, there was no corresponding shift in the control siRNA or vehicle-treated groups, indicating that neither the HiPerFect transfection reagent nor the nonspecific siRNA affected PTK7 expression. When PTK7 expression was probed after 12 h, 24 h, 30 h and 48 h of transfection using Western blot (Fig. 1B), the results clearly showed that the level of PTK7 expression decreased after 48 h of transfection. In addition, total mRNA was extracted from the untreated, vehicle, nonspecific siRNA, and PTK7 siRNA groups. As shown in Fig. 1C, PTK7 siRNA induced 75–80% reduction of PTK7 mRNA in HCT 116 cells. These results indicated that both PTK7 protein and mRNA expression levels were greatly decreased by PTK7 siRNA. This proved the function and efficiency of PTK7 siRNA and provided a solid basis for our study of PTK7's functional role.

### Viability and proliferation of PTK7 siRNA-treated HCT 116 cells

**Figure 2:**
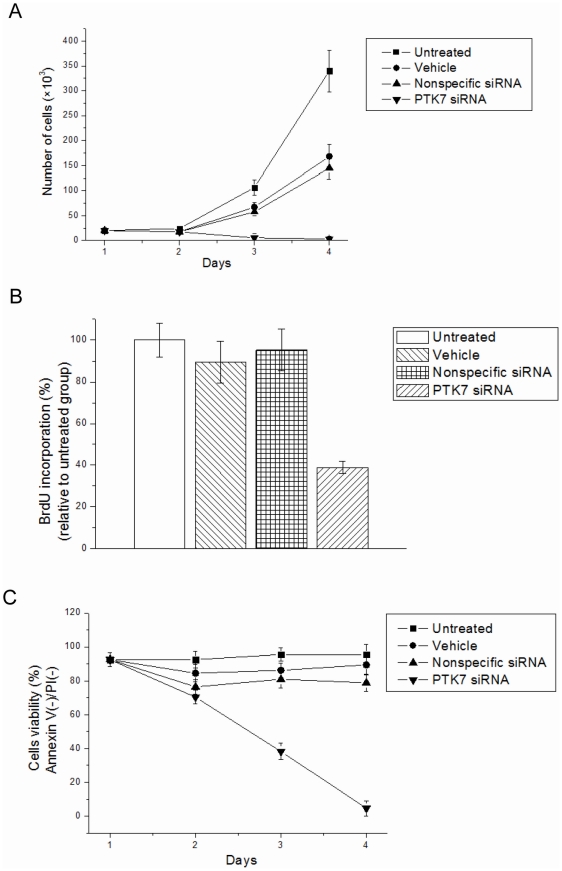
Cell viability in HCT 116 cells after treatment with vehicle, nonspecific siRNA and PTK7 siRNA. Data are mean±s.d. of three independent experiments. (A) The number of live cells was counted daily for 4 days using Trypan blue. (B) BrdU incorporation relative to untreated cells detected by flow cytometry. Cells were incubated with 10 µM BrdU for 2 h after 48 h of treatment. The amount of BrdU incorporation was normalized to the untreated group. Data are mean±s.d. of three independent experiments. *Student's t-test: P<0.05. (C) Apoptosis occurrence in HCT 116 cells detected by Annexin V/PI stain on days 1–4 after transfection. Cells stained negative for both Annexin V and PI were considered healthy.

The effect of PTK7 suppression on the viability of HCT 116 cells was investigated by counting the total number of live cells every day after transfection. As shown in Fig. 2A, the number of live HCT 116 cells transfected with PTK7 siRNA was shown to be significantly different from that of untreated groups on day 4. This finding demonstrated a significant inhibition of cell viability in the HCT 116 cells treated with PTK7 siRNA. To confirm that the decrease of cell viability resulted from suppression of PTK7, the same assays were carried out with HCT 116 cells transfected with nonspecific siRNA or treated only with vehicle. The results showed that the PTK7 siRNA-treated sample contained the smallest number of cells. Although vehicle-treated and nonspecific siRNA-treated cells had smaller cell numbers than untreated cells, there were significantly fewer cells in the PTK7 siRNA-treated sample.

To ascertain the effect of suppression of PTK7 on HCT 116 cell proliferation, a BrdU incorporation experiment was performed to measure DNA synthesis. After 48 h of transfection, cells were seeded in 24-well culture plates and were incubated with 10 µM BrdU for 2 h. Cells were then fixed, and BrdU incorporation was detected using a FITC-labeled anti-BrdU antibody (Fig. 2B). Silencing of PTK7 significantly inhibited BrdU incorporation in HCT 116 cells, suggesting a direct effect of PTK7 protein on HCT 116 cell proliferation.

### Increase of apoptosis of PTK7 siRNA-treated HCT 116 cells

An Annexin V/PI staining experiment was carried out to study the possibility that knocking down PTK7 could affect the apoptosis of HCT 116 cells. Phosphatidylserine (PS) is located in the inner leaflet of the cell membrane in healthy cells. During apoptosis, PS becomes translocated to the outer surface of the cell membrane, and Annexin V/PI assay detects the PS on the outer surface. The results in Figure 2C show that the PTK7 siRNA group showed significant increase in apoptotic cells on day 4 compared with untreated, vehicle, and nonspecific siRNA control groups.

### Changes in mitochondrial membrane potential and activation of caspase-9 in HCT 116 cells treated with PTK7 siRNA

**Figure 3:**
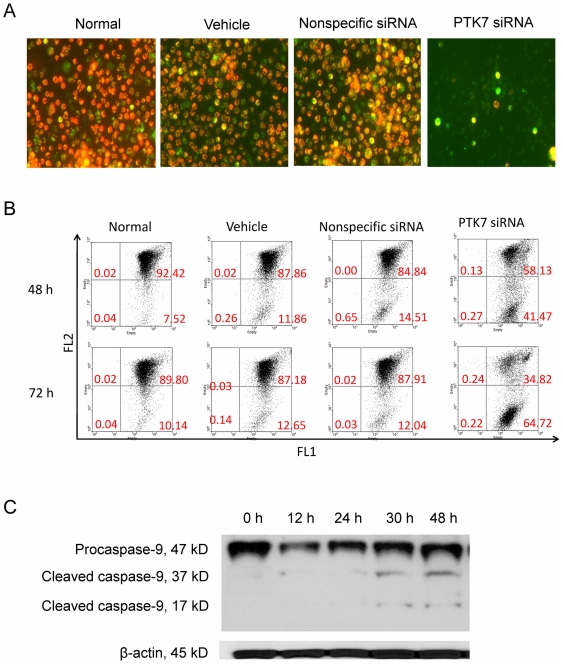
Involvement of mitochondrial pathway in apoptosis induced by PTK7 scilencing. (A) Fluorescence microscope detection of mitochondrial membrane potential in treated HCT 116 cells. (B) Flow cytometry detection of mitochondrial membrane potential in treated HCT 116 cells. (C) Activation of caspase-9 involved in apoptosis induced by knocking down PTK7. The membrane was stripped and reprobed by β-actin antibody, as a loading control.

To study the mechanism through which knocking down PTK7 induces apoptosis in HCT 116 cells, the effect of knocking down PTK7 on mitochondrial membrane potential (ΔΨ_m_) was determined by fluorescence microscopy (Fig. 3A) and flow cytometry (Fig. 3B). Dye JC-1 was used as an indicator. In healthy cells, polarized mitochondria have a negative charge, which allows JC-1 dye with delocalized positive charge to enter the mitochondrial matrix and accumulate there. When the critical concentration is exceeded, JC-1 forms J-aggregates, and the cells become fluorescent red (FL2). In apoptotic cells, the mitochondrial membrane potential collapses, and JC-1 cannot accumulate within the mitochondria. In these apoptotic cells, JC-1 remains in the cytoplasm in a green fluorescent monomeric form (FL1). After HCT 116 cells were transfected with siRNA or control as described above and incubated for 48 h or 72 h, the decrease of ΔΨ_m_ in HCT 116 cells transfected with PTK7 siRNA was observed. After 72 h of transfection, the percentage of cells with polarized mitochondria was 90%, 87% and 88% for cells in the untreated, vehicle and nonspecific siRNA groups, respectively. However, only 35% of total cells in the PTK7 siRNA group had polarized mitochondria. This trend was seen even at 48 h when only 58% cells had polarized mitochondria. These data suggested that mitochondrial dysfunction was involved in the apoptosis induced by PTK7 knockdown.

A variety of signalling pathways may be involved in apoptosis, and the mitochondria play a major role. Mitochondrial dysfunction causes the release of cytochrome *c*, which activates caspase-9, in turn fueling apoptosis. Caspase-9 activation was detected by Western blot after HCT 116 cells were transfected with PTK7 siRNA and cultured for 12 h, 24 h, 30 h, and 48 h, respectively. As shown in Figure 3C, caspase-9 was activated and involved in the apoptosis induced by PTK7 knockdown.

### Role of caspase-10 in PTK7-knockdown-induced apoptosis

**Figure 4:**
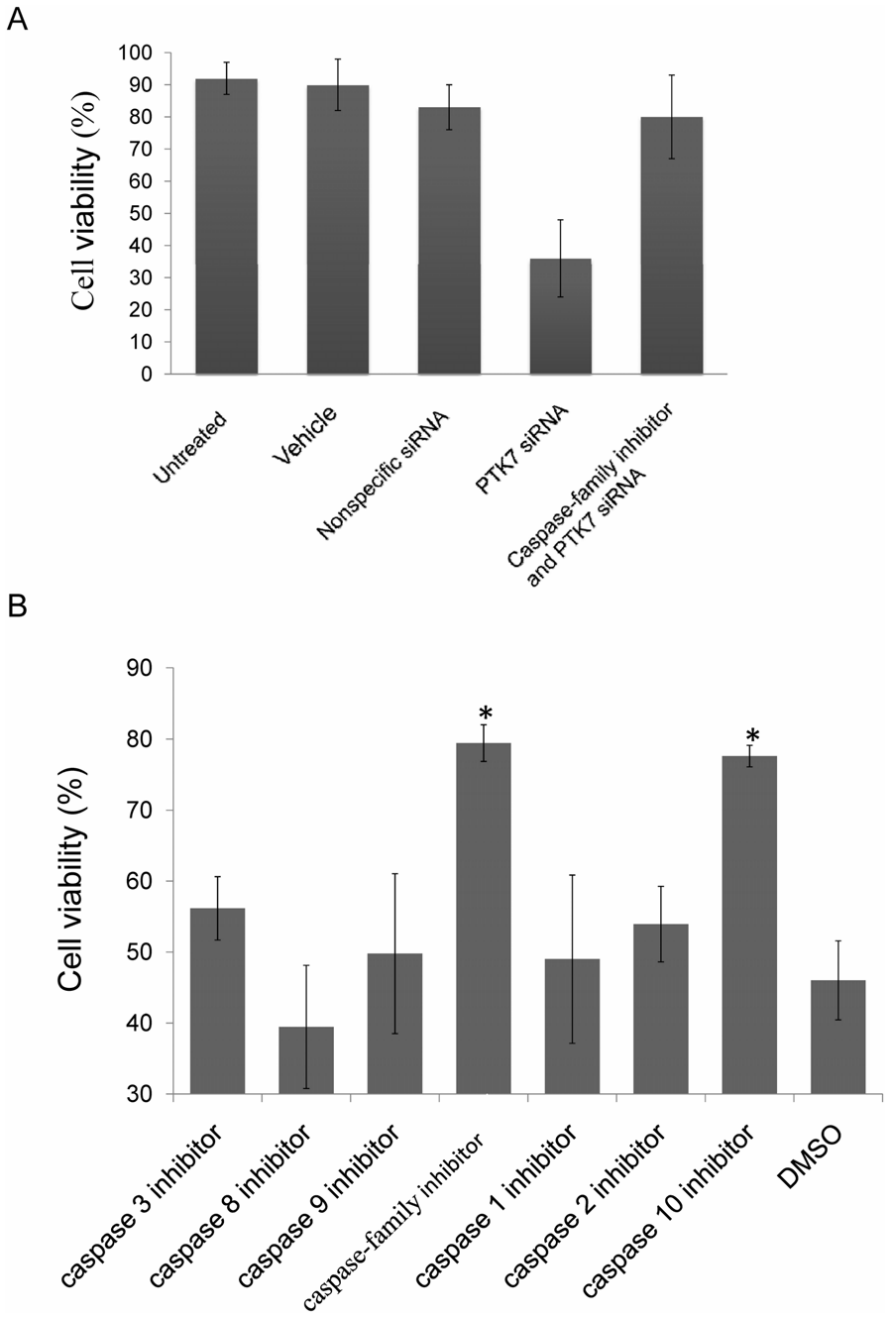
Cell viability after incubation with caspase inhibitors prior to transfaction of PTK7 siRNA. (A) Apoptosis induced by knocking down PTK7 was caspase-dependent. Data are mean±s.d. of three independent experiments. *Student's t-test: P<0.05. (B) Caspase-10 inhibitor totally blocked the apoptosis induced by knock down of PTK7. Data: mean±s.d. of three independent experiments, *Student's t-test: P<0.05.

To determine whether caspases mediate the apoptosis induced by knock down of PTK7, cells were pretreated with a pancaspase inhibitor or one of several single-caspase-specific inhibitors. Rescue of the cells from apoptosis would mean that the inhibited caspase was implicated in PTK7-deficient cell death. After pre-incubation of the HCT 116 cells with 20 µM pancaspase-family inhibitor at 37°C for 3 h, the cells were transfected with siRNA for 48 h. After incubation for 48 h, cell viability was tested using Annexin V/PI (Fig. 4A). Cells pre-incubated with pancaspase-family inhibitor showed good cell viability (80±13%) after transfection with PTK7 siRNA, within uncertainty of the cell viability of the nonspecific siRNA group (83±7.5%). Meanwhile, cells directly transfected with PTK7 siRNA had significantly lower cell viability (36±12%). Pancaspase-family inhibitor blocked caspase activity and also blocked apoptosis induced by knock down of PTK7, indicating that the apoptosis induced by knock down of PTK7 is caspase-dependent.

To investigate which caspase plays the critical role in this apoptosis, HCT 116 cells were pre-treated with caspase-9 inhibitor (Z-LEHD-FMK), caspase-3 inhibitor (Z-DEVD-FMK), caspase-8 inhibitor (Z-IETD-FMK), caspase-family inhibitor (Z-VAD-FMK), caspase-1 inhibitor (Z-YVAD-FMK), caspase-10 inhibitor (Z-AEVD-FMK), caspase-2 inhibitor (Z-VDVAD-FMK), or DMSO vehicle at 37°C for 3 h, followed by transfection with PTK7 siRNA. After incubation for 48 h, cell viability was tested by Annexin V/PI using flow cytometry. As shown in Fig. 4B, inhibition of caspase-10 blocked apoptosis, with 79±3% of cells viable. This meant that caspse-10 may play a critical role in the apoptotic pathway induced by knock down of PTK7.

**Figure 5:**
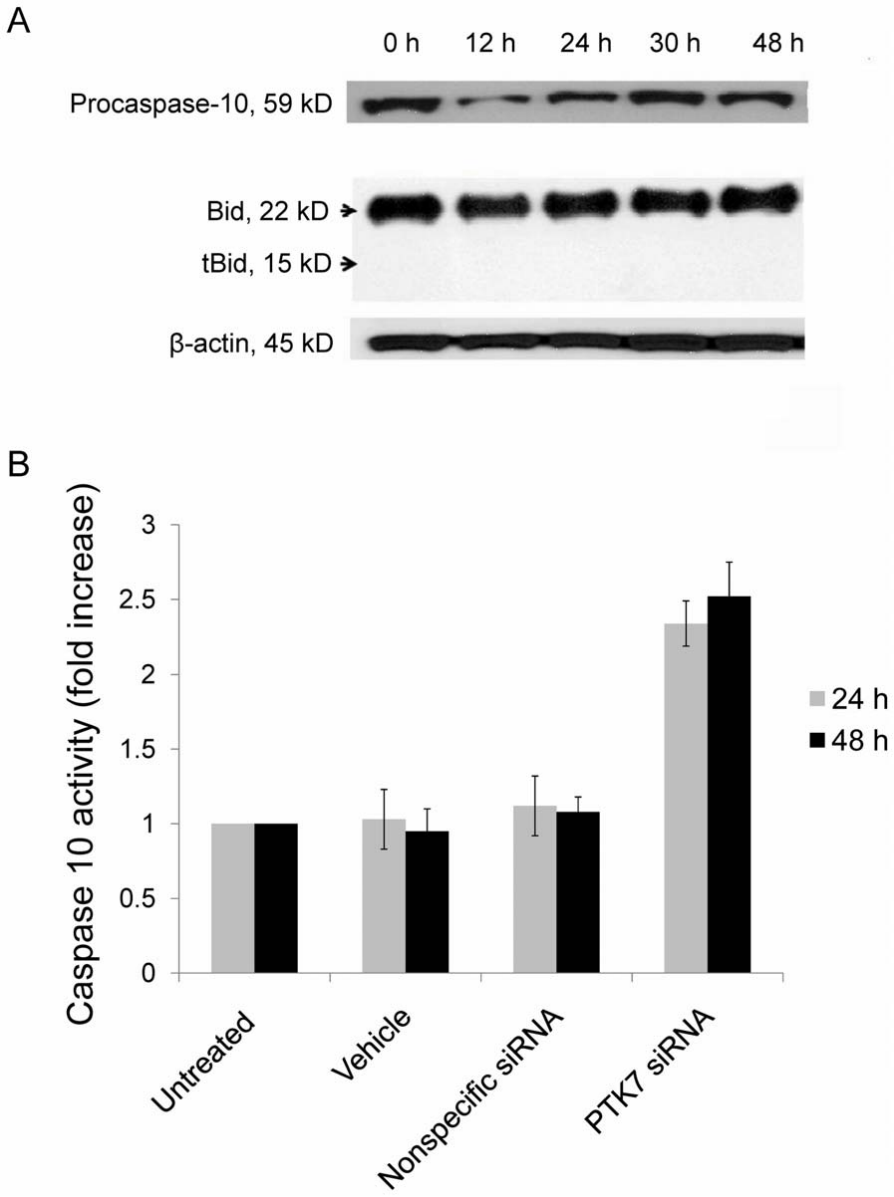
The activation of caspase-10 in apoptosis induced by knocking down of PTK7. (A) Western blot analysis of procaspase-10 and Bid in HCT 116 cells transfected by PTK7 siRNAs. The membrane was stripped and reprobed by β-actin antibody, as a loading control. (B) Caspase-10 activity in HCT 116 cells: untreated and treated with vehicle, nonspecific siRNA and siRNA. Results were given as ratios to caspase-10 activity in untreated cells. Data are mean±s.d. of three independent experiments. *Student's t-test: P<0.05.

To confirm the activation of caspase-10 in apoptosis induced by PTK7 knockdown, procaspase-10 protein levels in cell lysates transfected with PTK7 siRNA were examined using Western blot (Fig. 5A). Procaspase-10 decreased after 12 h of transfection and increased after 30 h of transfection. Also, as the linkage between the intrinsic pathway and the extrinsic pathway, the cleavage of Bid to tBid was investigated, and there was no obvious tBid, indicating that there was no signal transfer from the extrinsic pathway to the intrinsic pathway. In another experiment, PTK7 expression in HCT 116 was initially suppressed using PTK7 siRNA, resulting in apoptosis in these cells. The ability of cell lysates to cleave the peptide substrates (Ac-AEVD-AFC) was tested as an indicator of caspase-10 activity. The results in figure 5B show significant increase in caspase 10 activity in cells treated with PTK7 siRNA.

### Protein p53 involvement in PTK7-knockdown-induced apoptosis

**Figure 6:**
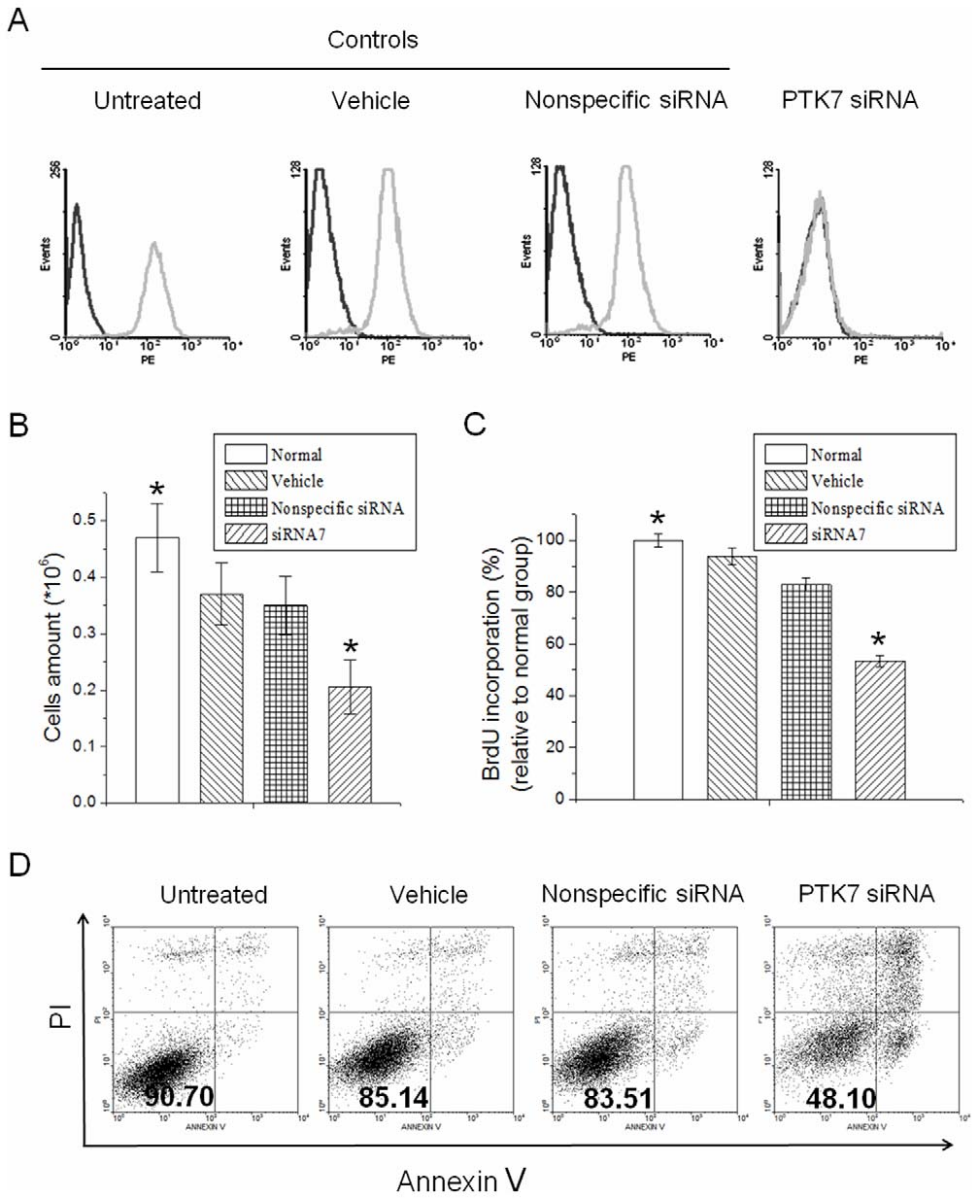
PTK7 expression and cell apoptosis induced by knocking down of PTK7 in p53-null HCT 116 cells. (A) Flow cytometry assay for the binding of the PE-labeled anti-PTK7 with p53-null HCT 116 cells (Grey curves). The black curves represent the background binding of anti-IgG-PE. The concentration of the antibody in the binding buffer was 2 µg/µL. (B) The number of live p53-null HCT 116 cells was counted on day 4 after treatment with vehicle, nonspecific siRNA and PTK7 siRNA. Data are mean±s.d. of three independent experiments. *Student's t-test: P<0.05. (C) BrdU incorporation relative to untreated cells detected by flow cytometry. p53-null HCT 116 Cells were incubated with 10 µM BrdU for 2 h after 48 h of treatment. The amount of BrdU incorporation was normalized to the untreated group. Data are mean±s.d. of three independent experiments. *Student's t-test: P<0.05. (D) Apoptosis occurrence in p53-null HCT 116 cells detected by Annexin V/PI stain on days 4 after transfection. Cells stained negative for both Annexin V and PI were considered healthy and percentage was shown in the figure.

Protein p53 has been proved as a tumor suppressor protein in humans[31], and HCT 116 cells express wide-type p53.[32], [33] In order to study the involvement of p53 in the apoptosis induced by PTK7 knockdown, p53-null HCT 116 was used as the second cell line to carry out PTK7 knockdown and other related experiments. First, the PTK7 expression level without/with siRNA treatment was monitored by flow cytometry (Fig. 6A). The p53-null HCT 116 cells express a high amount of PTK7 on the cell membrane, but after 48 hours of PTK7 siRNA transfection, the peak for anti-PTK7-PE in p53-null HCT 116 shifted back to the peak of the background control protein, anti-IgG-PE. This indicated that the PTK7 expression level in p53-null HCT 116 cells transfected with PTK7 siRNA was greatly decreased. Next, the number of live p53-null HCT 116 cells transfected with PTK7 siRNA was shown to be significantly different from that of untreated groups on day 4, demonstrating a significant inhibition of cell viability in p53-null HCT 116 cells by treating with PTK7 siRNA (Fig. 6B). And in a BrdU incorporation experiment, silencing of PTK7 significantly inhibited BrdU incorporation in p53-null HCT 116 cells, suggesting a direct effect of PTK7 protein on cell proliferation (Fig. 6C). On the other hand, the Annexin V/PI staining experiment showed that the PTK7 siRNA-treated p53-null HCT 116 showed significant increase in apoptotic and dead cells on day 4 compared with untreated, vehicle, and nonspecific siRNA control groups.

**Figure 7:**
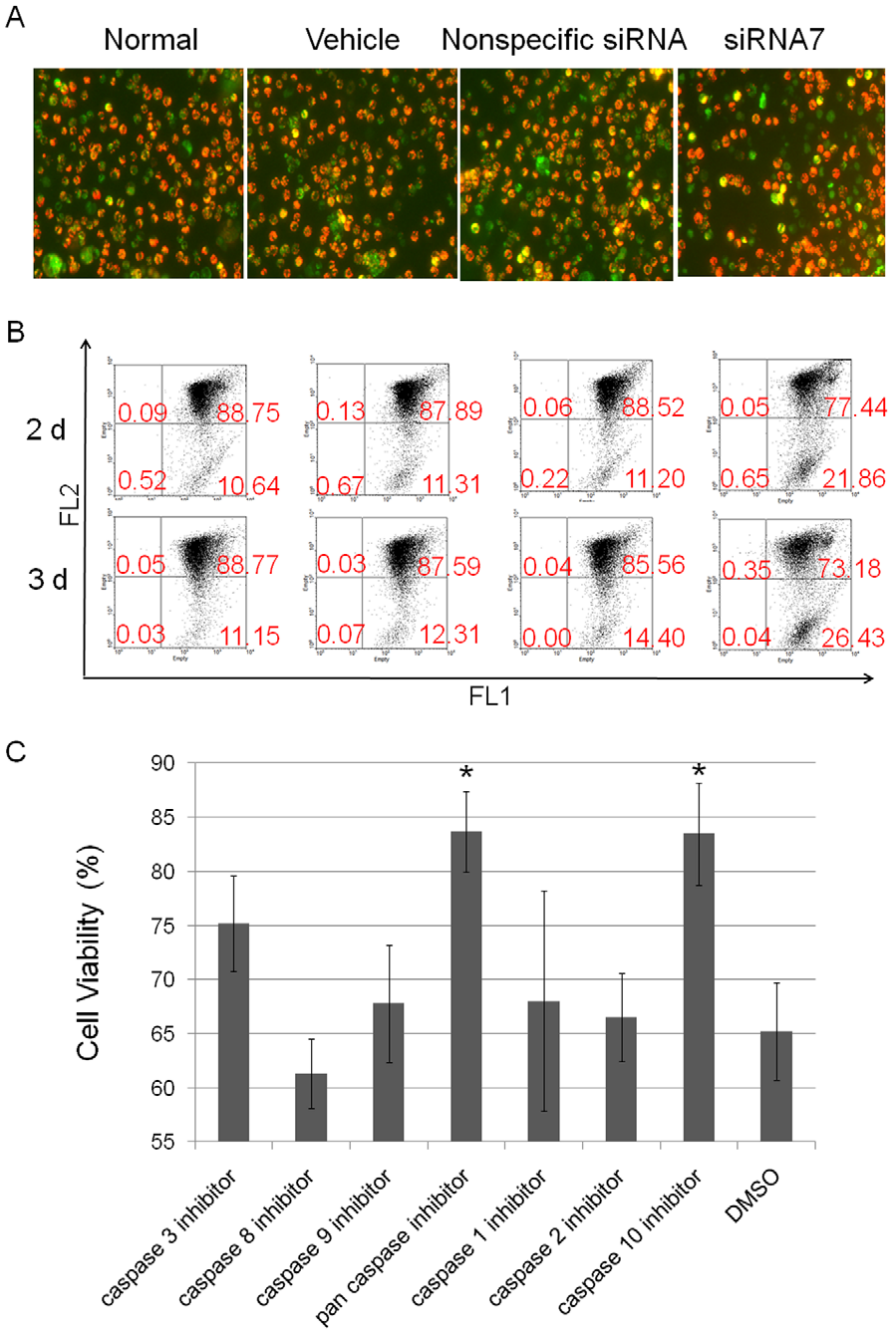
Mitochondria and caspase-10 involvement in the apoptosis induced by knocking down of PTK7 in p53-null HCT 116 cells. (A) Fluorescence microscope detection of mitochondrial membrane potential in treated p53-null HCT 116 cells. (B) Flow cytometry detection of mitochondrial membrane potential in treated p53-null HCT 116 cells. (C) Cell viability after incubation with caspase inhibitors prior to transfaction of PTK7 siRNA. Caspase-10 inhibitor totally blocked the apoptosis induced by knock down of PTK7. Data: mean±s.d. of three independent experiments, *Student's t-test: P<0.05.

The apoptosis induced by PTK7 knockdown has been proved to be caspase-10 dependent in wild type HCT 116. So the apoptosis pathway in p53-null HCT 116 induced by PTK7 knockdown was further investigated. JC-1 experiment was monitored by both fluorescence microscopy (Fig. 7A) and flow cytometry (Fig. 7B). Clearly, mitochondrial membrane potential decreased in p53-null HCT 116 cells treated with PTK7 siRNA. At the same time, a caspase inhibitor experiment was carried out using p53-null HCT 116 cells. As shown in Fig. 7C, pancaspase inhibitor or caspase-10 inhibitor treatment inhibited the apoptosis induced by PTK7 knockdown compared to all other inhibitors, which indicated the apoptosis in p53-null HCT 116 cells induced by PTK7 knockdown was caspased-10 dependent.

## Discussion

The present work demonstrates that RNAi suppression of PTK7 induces caspase-10-dependent apoptosis in both wild type and p53-null HCT 116 cells. Small interfering RNA is a very popular reverse genetics tool, which inhibits gene expression through sequence-specific degradation of target mRNA. This study showed that siRNA efficiently suppressed PTK7 expression at the level of both mRNA and protein. A nonspecific siRNA was used as a negative control to confirm that suppression of PTK7 was the result of the specific silencing effect of PTK7 siRNA.

After confirming suppression of PTK7 by siRNA, we then considered whether the inhibition of PTK7 would affect cell viability and proliferation. Trypan Blue Exclusion Assay showed that the number of live HCT 116 cells transfected with PTK7 siRNA was remarkably less than that of the control groups on day 4. Compared to nonspecific siRNA group as negative control, it was clear that suppression of PTK7 accounted for the inhibition of cell viability. To assess the effect of PTK7 knockdown on HCT 116 cell proliferation, a BrdU incorporation experiment was performed to measure DNA synthesis. Interestingly, PTK7 silencing significantly inhibited BrdU incorporation in HCT 116 cells, indicating that knock down of PTK7 expression had a direct effect on HCT 116 cell growth. In fact, PTK7 has been identified as a gene expressed in primary colon carcinoma, and overexpression of PTK7 is often found in colon carcinoma cells. Furthermore, knock down of PTK7 induced HCT 116 cell apoptosis, verified through Annexin V/PI stain. After knock down of PTK7, ratios of apoptotic HCT 116 cells revealed by Annexin V/PI stain showed a large increase of percentage of apoptotic HCT 116 cells. These results provide evidence that suppression of PTK7 can significantly increase the occurrence of apoptosis in HCT 116 cells, and that an excess of PTK7 can be associated with resistance of cancer cells to induction of cell death.

The results further demonstrated that knock down of PTK7 caused a large decrease in mitochondrial membrane potential of HCT 116 cells, suggesting that mitochondrial dysfunction may be involved in this apoptosis, and that the mitochondrial pathway to cell death may play an important role in apoptosis induced by knock down of PTK7. Caspase-9 was also activated after PTK7 siRNA treatment in HCT 116 cells. At the same time, apoptosis inhibition experiments showed that caspase-10 also plays a critical role in apoptosis induced by knock down of PTK7 in HCT 116 cells. Interestingly, caspase-8 inhibitor had no effect on this apoptosis at all, even though it has always been thought that caspase-8 and caspase-10 play identical roles in the extrinsic pathway to cell death. Western blot was used to examine the procaspase-8, -10 and active caspase-8 levels in PTK7 siRNA-treated HCT 116 cells. Procaspase-10 level changes were obvious, but active caspase-8 was not detectable (data not shown). Additionally, Bid/t-Bid level changes were examined, and no t-Bid was found (Fig. 5A) indicating that there was no signal transfer from the extrinsic pathway to the intrinsic pathway. Thus, the extrinsic pathway was not involved as reported by Filomenko P. et al.[20].

Furthermore, p53-null HCT 116 cells were used to study the involvement of p53 in the apoptosis induced by PTK7 knockdown. When treated with PTK7 siRNA, cell proliferation decreased and apoptosis increased in p53-null HCT 116 cells. Also, mitochondria were involved in the apoptosis, which was caspase-10 dependent. When comparing the results between wild type HCT 116 and p53-null HCT 116, PTK7 knockdown had less effect on cell proliferation and apoptosis in p53-null HCT 116 cells, but the apoptosis induced by PTK7 knockdown was caspase-10 dependent in both cell lines. Therefore, the effect of PTK7 knockdown on cell apoptosis was p53 related but not dependent.

Altogether, the results show that the knock down of PTK7 in wild type HCT 116 cells and p53-null HCT 116 cells induces cell apoptosis and affects cell proliferation. Also, caspase-10 activation plays a critical role in the caspase cascade, downstream of mitochondria after knock down of PTK7.

### Conclusion

In conclusion, suppression of PTK7 significantly increases apoptosis and inhibits cell proliferation in HCT 116 cells, indicating that PTK7 may play an important role in maintaining cancer cell viability. Apoptosis induced by knock down of PTK7 was caspase-10-dependent, and caspase-10 activation was downstream of mitochondria. Therefore, the use of PTK7 siRNA, or other methods that counteract PTK7 function, may be valuable in the development of cancer therapeutic agents.
